# A Retrospective Literature Evaluation of the Integration of Stress Physiology Indices, Animal Welfare and Climate Change Assessment of Livestock

**DOI:** 10.3390/ani11051287

**Published:** 2021-04-30

**Authors:** Edward Narayan, Michelle Barreto, Georgia-Constantina Hantzopoulou, Alan Tilbrook

**Affiliations:** 1 School of Agriculture and Food Sciences, Faculty of Science, The University of Queensland, St Lucia, QLD 4072, Australia; g.hantzopoulou@uq.net.au; 2 Centre for Animal Science, Queensland Alliance for Agriculture and Food Innovation, The University of Queensland, St Lucia, QLD 4072, Australia; a.tilbrook@uq.edu.au; 3 School of Veterinary Sciences, Faculty of Science, The University of Queensland, St Lucia, QLD 4072, Australia; m.barreto@uq.edu.au

**Keywords:** animal welfare, stress biomarkers, livestock production, climate change, future sustainability

## Abstract

**Simple Summary:**

Rapidly expanding global human population has led to increased supply chain demands on animal-based farming systems and the desire for environmentally friendly products. This has also resulted because of socio-political pressure and increased public concerns over the impacts of conventional agriculture on the environment. In order to be sustainable, animal production systems must also advance animal welfare, avoiding physically and psychologically stressful situations for the animals and apply innovative methods of reducing contribution of farming practices to global climate change while also functioning at optimum productivity. Consequently, to achieve a practical and effective improvement towards environmental sustainability, animal-based agriculture should consider animal welfare assessment, objective measures of physiological stress, climate change evaluation and animal productivity in a multi-dimensional and holistic approach.

**Abstract:**

In this retrospective study, we conducted a desktop-based analysis of published literature using the ScienceDirect™ search engine to determine the proportion of livestock research within the last 7 years (2015–2021) that have applied animal welfare assessment combining objective measures of physiological stress and evaluation of climate change factors in order to provide an account of livestock productivity. From the search results, 563 published articles were reviewed. We found that the majority of the literature had discussed animal production outcomes (*n* = 491) and animal welfare (*n* = 453) either individually or in conjunction with another topic. The most popular occurrence was the combination of animal welfare assessment, objective measures of stress physiology and production outcomes discussed collectively (*n* = 218). We found that only 125 articles had discussed the impact of climate change (22.20%) on livestock production and/or vice versa. Furthermore, only 9.4% (*n* = 53) of articles had discussed all four factors and published research was skewed towards the dairy sector. Overall, this retrospective paper highlights that although research into animal welfare assessment, objective measures of stress and climate change has been applied across livestock production systems (monogastrics and ruminants), there remains a shortfall of investigation on how these key factors interact to influence livestock production. Furthermore, emerging technologies that can boost the quantitative evaluation of animal welfare are needed for both intensive and extensive production systems.

## 1. Introduction

The United Nations Food and Agriculture Organization (FAO) has predicted that in order to supply the 9.1 billion people expected to inhabit the earth by 2050 global food production must increase by 70% [1]. Efforts to ensure food security have followed, but an increase in agricultural productivity is not the only piece in this complex puzzle. Prolonged droughts and natural disasters are two examples of extreme weather events induced by climate change, which threaten global agriculture [2]. Moreover, a reduction in water availability and climatic variability not only threaten crop yield but also impact on animal production [3].

Animal welfare can be defined as a state within the animal, what the animal is experiencing at any given time. It involves an integration of the biological responses elicited by an animal to their environment, and these responses can be indicated by changes in physiology, behaviour, health status, cognition and productive outputs [4,5]. Animal welfare is a multi-dimensional science, which is ever evolving as we find new ways to better understand non-human animals [6]. One way to objectively assess the welfare status of an animal is through measuring if the animal is under physiological stress, which is a biological response elicited when an animal is confronted with a threat to its wellbeing [7,8,9]. The consequences of chronic, severe or ongoing stress are known to have long term implications, since for example, stress hormones, such as cortisol, can hinder immune function and thus, the effectiveness of which an individual can fight diseases and pathogens [8,9,10,11]. Animal Welfare has received particular attention in livestock species due to an ethical obligation to maintain a humane environment for production animals, however productivity and welfare improvements are not always parallel [12,13]. The vast intensification of conventional agriculture has contributed to growing political, economic and public concern about sustainability issues within this [14]. As a result, animal agriculture is currently under a state of sustained pressure to meet welfare standards. These public concerns may stem from a belief that intensification and the increased productivity that follows it must come at a price of compromised animal welfare [15].

Efforts to reduce the contributions of livestock production to climate change have involved modifications in industry management practices, policy changes and the introduction of new improved technologies to monitor animal welfare and productivity (e.g., precision livestock monitoring), thus minimising wastage [16,17]. Since most of the expected human population growth will occur in developing countries where animal agriculture is a major source of income [1], the introduction of animal welfare guidelines and practices to developing countries is also crucial. However, this can be met with societal barriers such as cultural practices where animals are intensively handled for some cultural traditions.

It has been found that improving farm animal welfare can improve farm profitability and environmental sustainability [15,18]. For example, reducing stress and improving the welfare of livestock leads to lower mortality rates, better health and productivity, as well as superior immune response of livestock, which causes them to need less medication and it also reduces the risk of zoonotic and foodborne diseases [15,18]. As mentioned earlier, improving animal welfare can benefit the animal’s well-being, however it is not always the case that improvements in welfare will result in higher economic gain for the farmers and can be another barrier towards the successful introduction of practical animal welfare solutions in some industries [15,18].

By accounting and planning for climatic variability, farmers can prevent significant financial losses down the production line due to extreme climatic events such as heat waves and floods that would otherwise affect the reproductive output and productivity of their animals [19,20,21]. As mentioned, global climate change threatens livestock production. However, intensive animal production is also one of the highest contributors of greenhouse gasses particularly carbon dioxide, methane and nitrous oxide [22]. Finding ways to reduce agricultural greenhouse gas emission is currently a hot topic in scientific research and literature, however any mitigation technique to improve the environmental impact of animal agriculture needs to take animal welfare into account in order for it to be a valuable and realistic solution [23].

The primary aim of this retrospective paper is to investigate scientific articles published in recent years (2015–2021), in order to determine the proportion of livestock studies that have considered Animal Welfare (AW) and evaluation of physiological stress (S), climatic component (e.g., heat stress) (CC) and production outcomes (AP) (e.g., performance indicators) when reporting data, evaluating issues in production systems and/or proposing improvements. There are many other important facets of biological and production systems such as reproduction, immune function, behaviour and also economic analysis, however for the purpose of our study we focussed on AW, S, CC and AP to keep the interpretation and discussions straightforward. In particular, AW, S and CC which are most often appearing in media articles and debates about conventional animal agriculture.

## 2. Materials and Methods

### Non-Systematic Literature Search and Meta-Analysis

Two keyword-based searches were conducted on ScienceDirect™ (sciencedirect.com accessed on 18 March 2020 and 16 April 2021). This process identified peer-reviewed journal articles published in English between 2015–2021 that had investigated one or more of the following factors in livestock: (1) Animal Welfare (AW), (2) Stress (S), (3) Climate Change (CC) and (4) Animal Production (AP). Please note that AW constitutes a broad spectrum of phenotypic (morphometrics and behaviour) and environmental parameters while the stress (S) keyword was selected to evaluate studies that have measured some aspect of stress biology/stress physiology of the studied livestock species. Studies had to have a focus on farmed animals, so wild animals were outside the scope of this paper. The farm animals comprised of both traditional and emerging animals (monogastrics and ruminants) and diverse animal products (e.g., milk, meat, eggs, etc.). The following search command was used: (farm animal welfare AND/OR production AND/OR climate change AND/OR stress). This is primarily a retrospective analysis and not a systematic review, we appreciate that the keywords are not exclusive list of key factors however they will hopefully provide a useful dataset for this discussion platform. See supplementary file for primary data (File S1).

The search results were narrowed to research, review and data articles published within the last seven years (2015–2021). On the 18th March 2020 this search yielded 916 results on ScienceDirect™ and 338 on the 16th April, 2021. All 1254 articles were screened for relevance to the inclusion criteria. Ultimately, 737 articles were selected from this pool to be included in the dataset for this retrospective analysis. Articles that specifically investigated one animal species and class were grouped into one of the following categories: beef cattle, broiler chickens, dairy cows, dairy goats, layer hens. Articles investigating a non-specific species or class of animal that did not fit into any of the groups already mentioned were grouped into one of the following categories: cattle, farmed fish, goats, pigs, poultry, ruminants, emerging industries, sheep. Finally, articles that were investigating animal agriculture in general and did not focus on one specific species or class of animal or any of the groups already mentioned were grouped under “not specific”. All articles could only be grouped under one category of animal. Results are presented qualitatively using excel and all primary data are available in Supplementary File.

## 3. Results

### 3.1. Descriptive Results

From the sample size of 737 articles between 2015 and 2021, the most frequently discussed criteria were AW × S × AP at 33.4% (*n* = 246 papers) (Table 1). In contrast, the least commonly discussed criteria were AW × CC (*n* = 2 papers) and AW × S × CC (*n* = 2 papers) (Table 1). The total number of articles that looked at all four factors (AW × S × CC × AP) was 64 papers or 8.7%.

The classes of animals discussed were categorised into 14 groups. The most common class of animal investigated was dairy cows at 27.5% (*n* = 203) (Table 2).

### 3.2. Category Interactions

#### 3.2.1. Animal Welfare

Animal welfare (AW) was the second most mentioned factor in the literature after animal production (AP), at *n* = 522 and *n* = 652 respectfully. Animal welfare was most commonly discussed with Stress (S) × Animal Production (AP) (*n* = 246, 47.1%), and this was followed by AP alone (*n* = 127, 24.3%) and with all factors (*n* = 64, 12.3%) (Table 2). Articles discussing AW contributed to the top three most common criteria discussed (*n* = 437). Out of the articles that only mentioned one criterion alone, AW was the most common (*n* = 20). Dairy cows were the most commonly discussed class of animal with regard to AW (*n* = 148), and this was followed by pigs (*n* = 68) (Table 3).

#### 3.2.2. Stress

Stress (S) was the third most commonly mentioned factor in the literature (*n* = 485). Stress was mostly commonly discussed with Animal Welfare and Animal Production (AW × S × AP: *n* = 246, 50.7%), and this was followed by all four factors (AW × S × CC × AP: *n* = 64, 13.2%) and by AP (S × AP: *n* = 82, 16.9%) (Table 4). Dairy cows were the most commonly discussed class of animal concerning S (*n* = 122), and this was followed by layer hens (*n* = 56).

#### 3.2.3. Animal Production

Animal production (AP) was the most commonly discussed factor in the literature (*n* = 652). Animal production was most commonly discussed with animal welfare and stress (AW × S × AP: *n* = 246, 37.7%) and this was followed by animal welfare (AW × AP: *n* = 127, 19.5%) and stress (S × AP: *n* = 82, 12.6%) (Table 5). Dairy cows were the most commonly discussed class of animal concerning AP (*n* = 191), and this was followed by layer hens (*n* = 66).

#### 3.2.4. Climate Change

Climate change (CC) was the least discussed factor in the literature (*n* = 164). Climate change was most commonly discussed with all four factors (AW × S × CC × AP: *n* = 64, 39.0%), and this was followed by animal production (CC × AP: *n* = 36, 22.0%) and stress and animal production (S × CC × AP: *n* = 32, 19.5%) (Table 6). Articles discussing climate change contributed to the top four least commonly discussed factors (*n* = 10). Dairy cows were the most commonly discussed class of animal concerning climate change (*n* = 43), and this was followed by farmed seafood (*n* = 28).

## 4. Discussion

Our retrospective analysis of the published literature using ScienceDirect™ search engine pertaining the use of the terms (Animal Welfare-AW, Stress-S, Animal Production-AP and/or Climate Change-CC) indicates that in the last 7 years livestock researchers have mostly applied AP with AW and S while CC has been the least applied term used in published research. Furthermore, the dairy was the most studied livestock animal group used in research followed by pigs and farmed seafood, while goats were the least used animal group by research published in the last 7 years.

### 4.1. Animal Welfare

As global population increases, and is expected to increase at an unprecedented rate over the next few decades, the animal industry must find new ways to ensure an uninterrupted quality food supply [24]. Outbreaks of production related diseases, such as Avian Influenza and Foot and Mouth Disease over the last two decades, have highlighted the gravity of good welfare management for the security of food safety, supply of disease-free farm animals [25].

A widely used model for the evaluation of animal welfare is the Five Domains, which places welfare challenges and opportunities into five overlapping categories (nutrition, environment, health, behaviour, and mental state), and is commonly used to evaluate welfare within applied contexts, such as farms [26]. The Five Domains model expanded on the five freedoms paradigm and aimed to facilitate more structured assessments of welfare [27]. There is a consensus that animals should not simply be able to cope with their environment, but should have opportunities for positive experiences in each of the Five Domains [26]. In contemporary animal welfare science, animal sentience is commonly acknowledged and valued in welfare assessments [28]. Animal sentience is the basis on why animals can experience positive and negative states, and therefore, the basis of why animal welfare assessments are important [29,30]. That being said, not all studies investigating animal welfare in livestock production systems that we have come across have meaningfully integrated the subjective emotional experience of animals in animal welfare discussions or evaluations. Studies in production animals frequently focussed on productivity indicators of welfare, such as low mortality, low disease prevalence or good feed intake, and not the animals’ cognitive or emotional experience. This allows for the approval of many of the routine procedures carried out on farms. For example, dehorning is a procedure that is routine across many dairy and beef farms, which involves the surgical removal of the horns [31]. Dehorning is an inarguably very painful and unpleasant procedure for the animal, and it is most commonly performed on farms with no pain relief such as anaesthesia or analgesia [32,33] in the USA, Europe and most other parts of the world [34]. Dehorning, albeit inarguably painful, results in fewer horn related injuries both within livestock and between the animals and people. Furthermore, most farmers believe the animals fully recover within days after the procedure, as was highlighted in a 2016 study [33]. However, there is a lack of objective studies that have investigated chronic pain or stress responses to dehorning [35], which negatively impacts welfare and the animal’s emotional wellbeing. Comparative studies are available in wildlife animal model (e.g., dehorning of game Rhinos; [36]).

Currently, the development of new-age technologies (e.g., remote sensing technology for reproductive and general health and well-being; artificial intelligence, [37] for the assessment of animal welfare and positive affective states (cognitive emotional well-being) has allowed animals to be assessed with greater ease than ever before [38].

### 4.2. Animal Stress

Stress and its relation to animal welfare has been a topic of dispute in scientific literature. Some authors believe that objective measurements of stress, more specifically those studies which base welfare assessments on the measurement of cortisol alone, lack scientific robustness as an evaluation of welfare [39,40,41]. In animal production it is important to assess stress responses of animals because the variation in stress responses can have important consequences for welfare, reproduction and production of animals [40,41]. This is because the short-term release of cortisol is a functional process which does not always signpost suffering or poor welfare and is rather essential for survival [7,42]. Moreover, the traditional methods of cortisol measurement can be invasive, and the alternatives for livestock industry use are experimental and scarce [43]. In this perspective, stress in livestock is more closely related to consequences in productivity rather than in welfare [44]. Since stress is an essential component of survival it is unlikely that stress could ever be completely eliminated in production animals [45]. However, in the case of farm animals, husbandry management should ensure that the causes of stress are minimized and where acute stressors (e.g., transportation) is unavoidable, the effects on the animal such as ill health must be attended to and remedied. As with welfare, the evaluation of stress should not rely on a single indicator and rather incorporate a combination of indicators [46]. To better understand which indicators are appropriate it is important to understand the potential causes of stress. The causes of stress can be social, such as social isolation [47] or housing with unfamiliar conspecifics [48] and/or environmental, such as thermal [49] or oxidative stress [50]. The effects of these stressors are species specific, meaning some species can be more prone to a stressor than another. For example, pigs are highly prone to heat stress during transport because pigs have adapted to lose body heat by wallowing, and it is usually not possible to perform this behaviour during transport [51]. The effects of stressors can also vary within species with regard to life-history and developmental stages. For example, dairy cows in early lactation are more prone to oxidative stress and stress related illnesses compared to cows in a late stage of pregnancy [52]. Although environmental stress is not limited to thermal causes, the implications of heat stress are the most reported on due to the severity of impacts on animal productivity [53]. The instances of heat stress in animal agriculture are expected to increase along with rising global temperatures. Minimally invasive biomarkers of acute and chronic stress such as faecal, hair and wool cortisol measurements are available and provide useful contributions to livestock health and welfare evaluation studies [54,55,56].

### 4.3. Climate Change

In 2010, greenhouse gas emissions from animal agriculture were responsible for 23% of the total modelled warming of 0.81 °C, which made the industry the largest anthropogenic contributor of emissions worldwide [57]. Although animal production is a heavy contributor to climate change, the effects of global warming on production are equally as dire [58]. Climate change poses serious threats to livestock production indicators such as water and feed availability, product yield and reproductive performance, animal and crop biodiversity and animal health and well-being [17]. As with animal welfare, the intensification of animal agriculture has ignited public and government concern over its effects on climate change. The intensification of existing agricultural land has partly been an alternative to the expansion of farmland into other ecosystems [59] and both scenarios have been equally as contentious [60]. These efforts were highlighted in a 2012 study which reported that the earths total agricultural land, comprised mostly of grasslands, had not increased in size since 1991 [61].

With climate change variability, comes extreme changes in ecological functions at the local level such as through prolonged droughts and unprecedented bushfires. Thus, climate change brings on-going psycho-social and financial challenges to farmers [62] in regional and rural areas through increased costs and losses associated with feeding stock, diseases and finding new ways for mitigating immediate effects of factors such as heat stress [63].

### 4.4. Animal Production

Animal meat production must nearly double by 2050 in order to feed the world’s population [64]. Currently, ruminants such as cattle (*Bos indicus* and *Bos Taurus*), sheep (*Ovis aries*) and goats (*Capra aegagrus hircus*) constitute most of the world’s agricultural land [65]. Dairy cow and beef cattle production are the two primary industries that makeup ruminant production [65]. Non-ruminant livestock such as chicken (*Gallus gallus domesticus*) and pig (*Sus scrofa domesticus*) farming have steadily increased in numbers over the last few decades [65]. Production can be measured by productive outputs such as milk, eggs, fibres such as wool or cashmere, and lean body mass. Growth and reproductive performance are also a way to measure productivity, and production can also be measured by the economic vitality of the animal product. For example, the assurance of food safety and consumer willingness to purchase are essential for farm and industry profitability and therefore, measurements of production. As an industry which provides income for over 1.3 billion people [16], the profitability of animal production is essential to its existence.

Animal production in developed countries has been improved largely through direct intervention of animal welfare frameworks (e.g., 5 freedoms and 5 domains), and there has also been strong research focus in areas of pain management [66,67]. However, developing countries in Asia and Africa are struggling to balance animal welfare objectives with animal production and profitability and issues such as access to space and feed remains today [68] and highlighted the need for more research programs focussed on improving livestock disease management and applying education and research to improve such as lowered stress and improvements in livestock nutrition.

## 5. Conclusions

It is highly encouraging to know that researchers have applied key themes such as Animal Welfare, Stress and Animal Production in combination when studying their livestock animal system. This could possibly be an indicator of the increased public concerns regarding the animal welfare issues in livestock production, and this increased public awareness has increased governmental agencies to direct more funds and policy actions in support of animal welfare domains such as animal sentience. It would also be unsurprising that more research is directed towards improving animal welfare rather than just minimising animal harms [69]. It is timely that livestock production research providers focus on a more well-rounded approach to solving the challenges related to animal production and welfare, and there certainly needs to be more research towards understanding how livestock animals respond to climate change. Previous research has focussed mainly on improving production traits, however in recent times there are strong foundations for more all-inclusive research approaches that integrate the themes of AW, AP, S and CC. Overall, by taking a more holistic approach we can understand whether productivity gain is creating an underlying imbalance in these other key areas. Such a discrepancy could potentially already be responsible for hidden costs that have the potential to cause major disruptions to the supply chain in the event of unprecedented climatic variability, such as prolonged drought and exposure to novel infectious diseases.

## Figures and Tables

**Table 1:** Column graphs showing number of articles that included one or more of the key factors factor (Animal Welfare—AW, Stress—S, Climate Change—CC, Animal Production—AP) in their research between 2015–2021.

Number of Criteria per Year								
Keywords	2015	2016	2017	2018	2019	2020	2021	Total
AW × S × AP	24	35	37	46	56	26	22	246
AW × AP	7	17	13	21	31	24	14	127
S × AP	6	4	4	11	7	33	13	78
AW × S × CC × AP	6	6	6	15	14	9	8	64
AP	2	1	1	3	4	15	17	43
AW × S	6	6	3	3	14	7	1	40
CC × AP	4	2	2	5	6	11	6	36
SS × CC × AP	3	2	2	2	9	9	4	31
AW	2	1	2	6	6	4	2	23
AW × CC × AP	1	1	4	5	5	4	1	21
S	0	2	1	1	2	5	6	17
S × CC	0	0	0	0	2	2	0	4
CC	0	1	0	2	0	0	0	3
AW × CC	0	0	0	0	0	1	1	2
AW × S × CC	0	1	0	0	0	0	1	2
Total	61	79	75	120	156	150	96	737

**Table 2:** Number of articles per class of animals and year. Goats included articles investigating goats in general with no focus on dairy, meat or fibre production. Ruminants included articles investigating ruminant production in general with no focus on any particular species or animal product.

Class of Animal	2015	2016	2017	2018	2019	2020	2021	Total
dairy cows	15	19	21	37	50	44	17	203
not specified/other	9	7	15	5	12	29	28	105
pigs	6	12	8	18	17	15	7	83
farmed seafood	3	4	7	12	17	20	15	78
broiler chickens	3	5	9	9	10	12	4	52
sheep	8	5	5	6	8	9	4	45
layer hens	2	7	2	7	13	3	3	37
beef cattle	4	2	1	5	6	9	4	31
cattle	0	4	1	7	3	2	6	23
emerging industries	5	3	0	7	6	2	0	23
poultry	1	2	3	3	4	4	6	23
dairy goats	1	3	3	2	4	0	1	14
ruminants	2	3	0	2	3	1	0	11
goats	2	3	0	0	3	0	1	9
Total	61	79	75	120	156	150	96	737

An article (*n* = 1) investigating sheep and goats together was also included in this category. Cattle included articles focusing on cattle production in general with no focus on animal product such as dairy or meat. Poultry included articles investigating poultry production in general with no focus on animal product such as eggs or meat. Articles investigating turkey (*n* = 5) and ducks (*n* = 2) were also included in this category. Emerging industries included articles investigating non-traditional farmed species of animals, and this category included buffalo (*n* = 7), mink (*n* = 7), meat rabbits (*n* = 4), camelids (*n* = 2), guinea pigs (*n* = 1), deer (*n* = 1) and crocodile (*n* = 1). Sheep included articles focusing on sheep production in general with no focus on animal product such as fibre or meat. Not specified included articles investigating animal agriculture in general which did not focus on one specific species or class of animal and did not fit into any of the other groups. Articles investigating broiler chickens and pigs (*n* = 3) were also included in this category. Fish included all articles investigating any farmed fish species within fish farming and aquaculture production. Articles investigating shrimp and crustacean production were also included in this category.

**Table 3:** Summary of articles discussing animal welfare (AW) interaction with Stress—S, Climate Change—CC, Animal Production—AP) in their research between 2015–2021.

Animal Group	AW	AW × S	AW × CC	AW × AP	AW × S × CC	AW × S × AP	AW × CC × AP	AW × S × CC × AP	Total
dairy cows	5	3	0	53	0	58	6	23	148
pigs	6	8	0	12	0	35	3	4	68
not specified	3	2	1	14	0	21	7	11	59
broiler chickens	3	4	0	17	0	20	1	1	46
farmed seafood	0	2	0	5	0	23	1	9	40
sheep	0	6	0	5	1	19	0	2	33
layer hens	1	3	0	5	0	12	2	3	26
beef cattle	0	2	0	3	0	11	1	3	20
emerging industries	2	3	0	1	0	12	0	1	19
cattle	0	1	0	3	0	13	1	0	18
poultry	0	2	0	6	0	9	0	0	17
dairy goats	0	2	0	2	0	8	0	0	12
ruminants	0	1	0	0	0	4	0	4	9
goats	0	1	0	1	1	1	0	3	7
Total	20	40	1	127	2	246	22	64	522

**Table 4:** Summary of articles discussing Stress (S) interaction with Animal Welfare—AW, Climate Change—CC, Animal Production—AP) in their research between 2015–2021.

Animal Group	S	AW × S	S × CC	S × AP	AW × S × CC	AW × S × AP	S × CC × AP	AW × S × CC × AP	Total
dairy cows	3	3	0	27	0	58	8	23	122
layer hens	2	8	0	6	0	35	1	4	56
emerging industries	9	2	0	6	0	23	5	9	54
not specified/other	0	2	0	12	1	21	7	11	54
sheep	0	6	0	6	0	19	2	2	35
broiler chickens	0	4	0	4	0	20	1	1	30
goats	0	3	0	7	0	12	2	3	27
beef cattle	1	2		1	0	11	2	3	20
ruminants	0	3	0	4	0	12	0	1	20
cattle	0	1	0	3	0	13	0	0	17
pigs	0	2	0	3	0	9	2	0	16
farmed seafood	0	1	4	3	1	1	0	3	13
poultry	0	1	0	0	0	4	2	4	11
dairy goats	0	2	0	0	0	8	0	0	10
Total	15	40	4	82	2	246	32	64	485

**Table 5:** Summary of articles discussing Animal Production (AP) interaction with Animal Welfare—AW, Climate Change—CC, Stress (S) in their research between 2015–2021.

Animal Group	AP	AW × AP	S × AP	CC × AP	AW × S × AP	AW × CC × AP	S × CC × AP	AW × S × CC × AP	Total
dairy cows	11	53	27	5	58	6	8	23	191
not specified	14	14	12	13	21	7	7	11	99
layer hens	2	12	6	3	35	3	1	4	66
emerging industries	5	5	6	8	23	1	5	9	62
broiler chickens	1	17	4	0	20	1	1	1	45
sheep	3	5	6	1	19	0	2	2	38
goats	2	5	7	0	12	2	2	3	33
beef cattle	2	3	1	4	11	1	2	3	27
cattle	0	3	3	2	13	1	0	0	22
pigs	1	6	3	0	9	0	2	0	21
ruminants	0	1	4	0	12	0	0	1	18
dairy goats	2	2	0	0	8	0	0	0	12
poultry	0	0	0	0	4	0	2	4	10
farmed seafood	0	1	3	0	1	0	0	3	8
Total	43	127	82	36	246	22	32	64	652

**Table 6:** Summary of articles discussing Climate Change—CC interaction with Animal Welfare (AW), Stress (S), Animal Production—AP) in their research between 2015–2021.

Animal Group	CC	AW × CC	S × CC	CC × AP	AW × S × CC	S × CC × AP	AW × CC × AP	AW × S × CC × AP	Total
dairy cows	1	0	0	5	0	8	6	23	43
not specified	0	1	0	13	1	7	7	11	40
farmed seafood	0	0	4	8	1	5	1	9	28
beef cattle	1	0	0	4	0	2	1	3	11
pigs	0	0	0	3	0	1	3	4	11
layer hens	0	0	0	0	0	2	2	3	7
ruminants	0	0	0	0	0	2	0	4	6
sheep	0	0	0	1	0	2	0	2	5
broiler chickens	0	0	0	0	0	1	1	1	3
cattle	0	0	0	2	0	0	1	0	3
goats	0	0	0	0	0	0	0	3	3
poultry	1	0	0	0	0	2	0	0	3
emerging industries	0	0	0	0	0	0	0	1	1
dairy goats	0	0	0	0	0	0	0	0	0
Total	3	1	4	36	2	32	22	64	164

## Data Availability

All primary data are available in the supplementary folder.

## Supplementary Materials

The following are available online at https://www.mdpi.com/article/10.3390/ani11051287/s1. File S1: Primary articles used for this review paper.
